# Supplementary material for: Gene Expression Profiling of Different Huh7 Variants Reveals Novel Hepatitis C Virus Host Factors
Source: Viruses. 2019 Dec 28;12(1):36. doi: 10.3390/v12010036 (PMC7019296; doi:10.3390/v12010036)
Supplement: Supplementary file 1 [file viruses-12-00036-s001.zip › Figure S1-10 and Table S1.pdf]

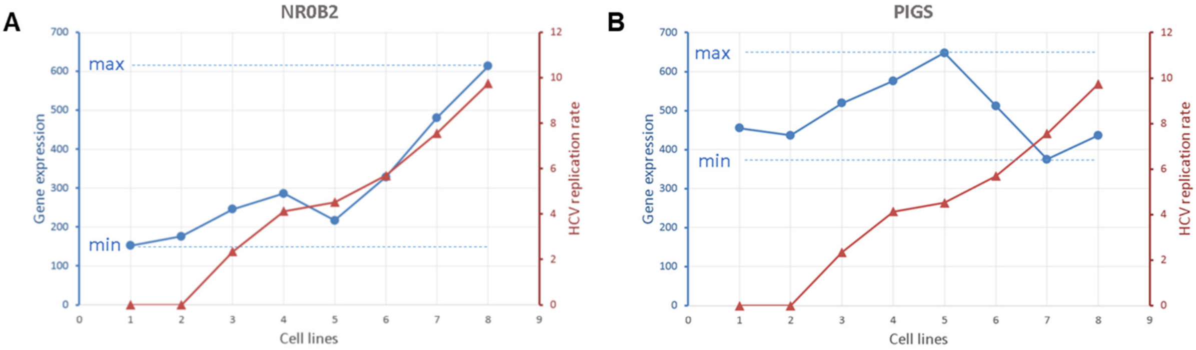

C

(1) Huh-7 p13 (2) Huh-7 p28 (3) Huh7-Lunet NT (4) Huh-7 p163 (5) Huh-7 p154 (6) Huh-7/5-2 p44 (7) Huh7-Lunet I (8) Huh7-Lunet II

Low HCV replication rate — High HCV replication rate

D

Gene ID	Symbol	Huh-7 p13	Huh-7 p28	Huh7-Lunet NT	Huh-7 p163	Huh-7 p154	Huh-7/5-2 p44	Huh7-Lunet I	Huh7-Lunet II	Accepted
1	A1BG	71	47	385	111	228	66	630	1278	yes
255022	CALHM1	29	30	35	32	41	37	37	36	yes
340267	COL28A1	29	42	31	31	46	30	28	49	yes
1296	COL8A2	29	28	33	33	33	31	35	34	no
1428	CRYM	65	67	124	105	120	124	131	177	yes
1556	CYP2B7P1	28	27	37	27	31	37	37	40	no
56605	ERO1LB	29	34	96	55	47	32	32	42	yes
2295	FOXF2	19	22	25	25	27	16	36	44	yes
29999	FSCN3	28	24	24	29	26	28	29	28	no
11318	GPR182	28	30	30	30	29	32	30	28	yes

High significance of gene expression measurements (P, M)

Low significance of gene expression measurements (A)

E

Gene pre-filtering criterions:

1. $\frac{\min(GE\_high\_HCV)}{\max(GE\_low\_HCV)} > 1.0$
2. $\frac{\max(GE\_high\_HCV)}{\max(GE\_low\_HCV)} > tsh$

Gene selection criterions:

1. Significant Pearson corr. betwen **incomplete** patterns of GE and HCV
2. Significant Spearman corr. betwen **incomplete** patterns of GE and HCV
3. Significant Pearson corr. betwen **complete** patterns of GE and HCV
4. Significant Spearman corr. betwen **complete** patterns of GE and HCV
5. max(GE_high_HCV)/max(GE_low_HSV) treshold: **tsh** = 2.0

Figure S1. Overview of gene selection procedure. (**A**, **B**) Two exemplary genes with either high (NR0B2) or low (PIGS) correlation between gene expression and HCV replication in eight different Huh7 cell lines. (**C**) Huh7 cell lines with low and high HCV replication rate that were used for gene expression profiling. (**D**) Examples of complete (eight P/M) and incomplete (less than eight P/M) patterns of gene expression measurements in eight Huh7 cell lines with indication of their acceptance or non-acceptance for subsequent correlation analysis. (**E**) Criterions of gene pre-filtering and selection for subsequent experimental evaluation. GE = gene expression; tsh = threshold.

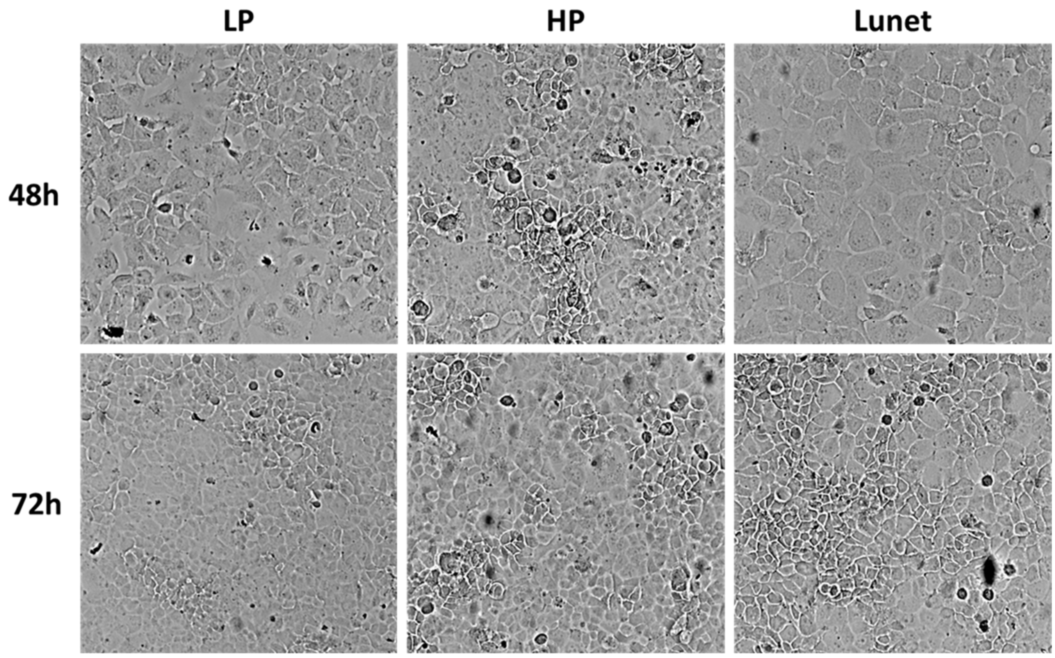

Figure S2. Huh7 cell lines with different HCV permissiveness show similar morphology. Huh7-LP (LP), -HP (HP), or -Lunet (Lunet) cells were seeded on cover slips, fixed after 48 or 72 hours and imaged.

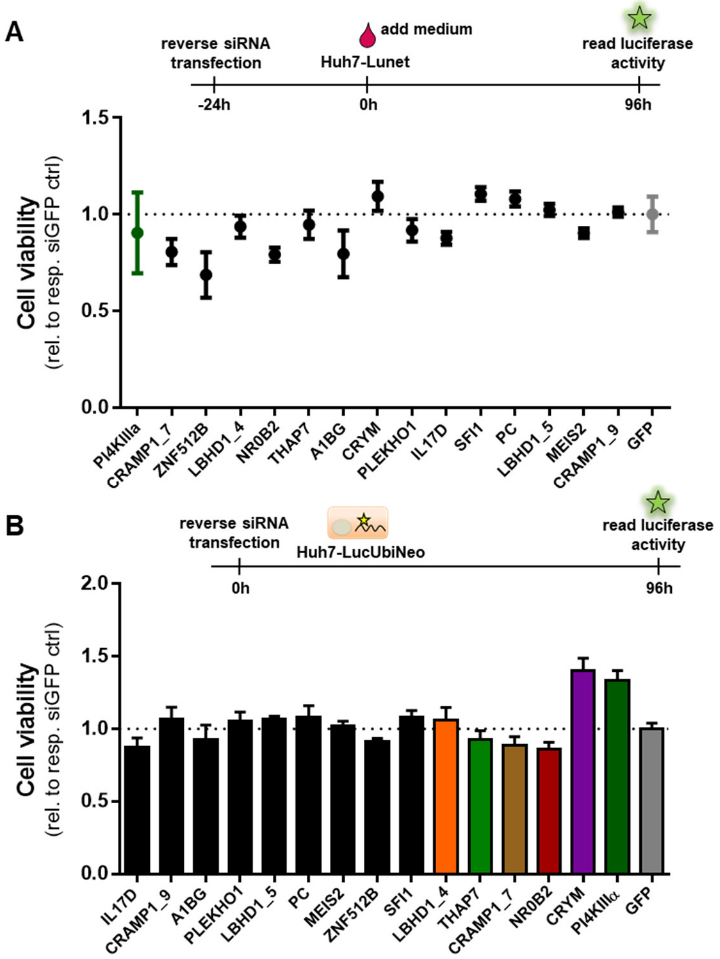

Figure S3. Cell viability assessment after siRNA-mediated knockdown of potential host factors and HCV infection (A) or in a stable replicon model (B) of highly permissive cells. (**A**) Huh7-Lunet cells were reverse transfected with siRNAs against target genes and cell viability was measured after 96 hours. Data is normalized to a control siRNA targeting GFP for each experiment individually. Shown are means and standard deviations from two independent biological experiments. (**B**) Huh7 cells with a stably replicating luciferase reporter HCV subgenome (gt2a) in the cytoplasm (Huh7-LucUbiNeo) were reverse transfected with siRNAs against target genes and cell viability was measured after 96 hours. Data is normalized to an siRNA targeting GFP and shown are means and standard deviations from two independent biological experiments.

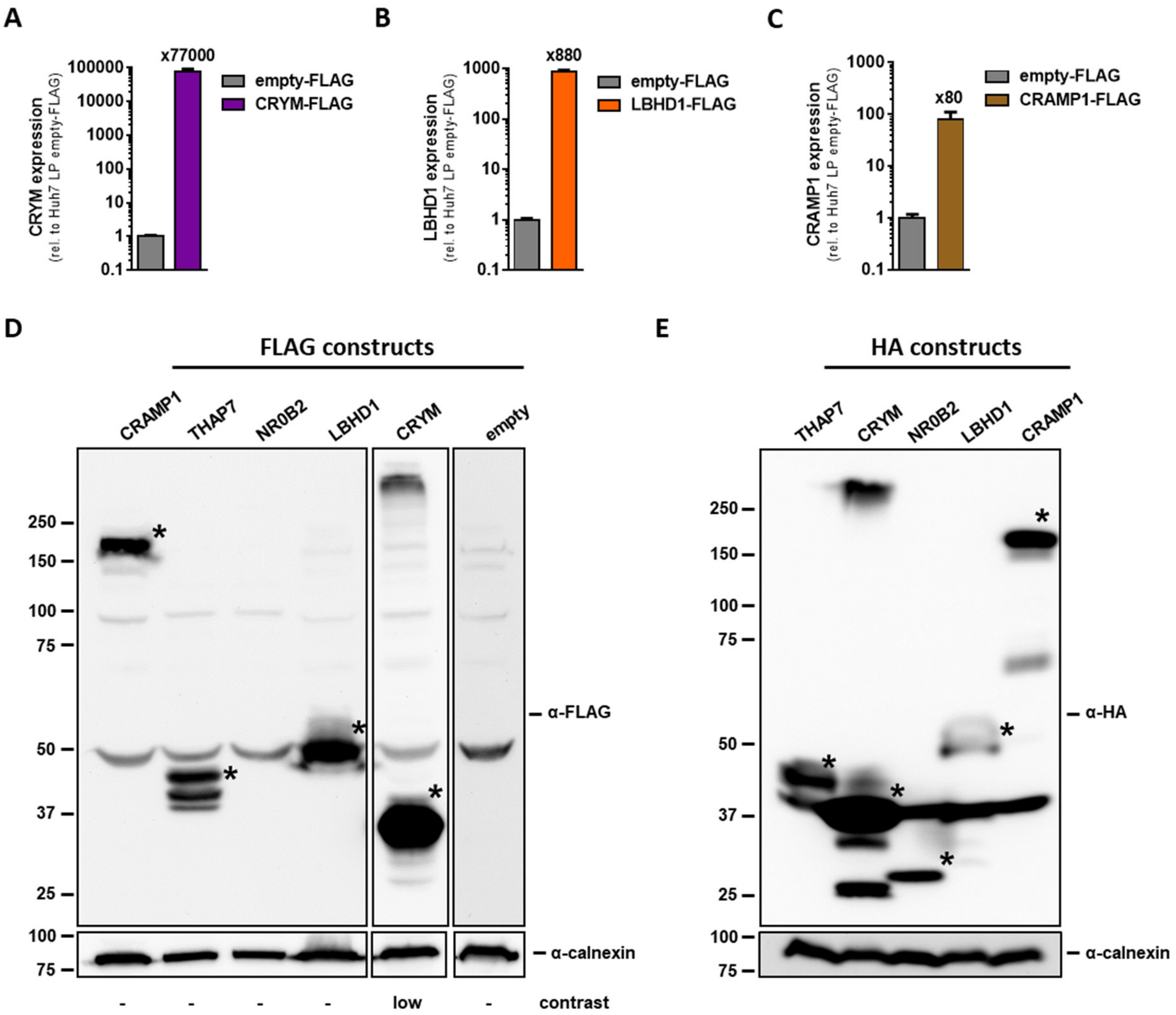

Figure S4. Putative host factors are robustly overexpressed in Huh7-LP cells. (**A**-**C**) Expression levels of CRYM (**A**), LBHD1 (**B**), and CRAMP1 (**C**) mRNA determined by qRT-PCR in the respective host factor-FLAG overexpressing Huh7-LP cells versus cells transduced with an empty-FLAG vector. (**D**, **E**) Immunoblots against host factor epitopes FLAG (**D**) or HA (**E**). Asterisks indicate bands corresponding to correct host factor size. Note the lower contrast for CRYM in panel (**D**).

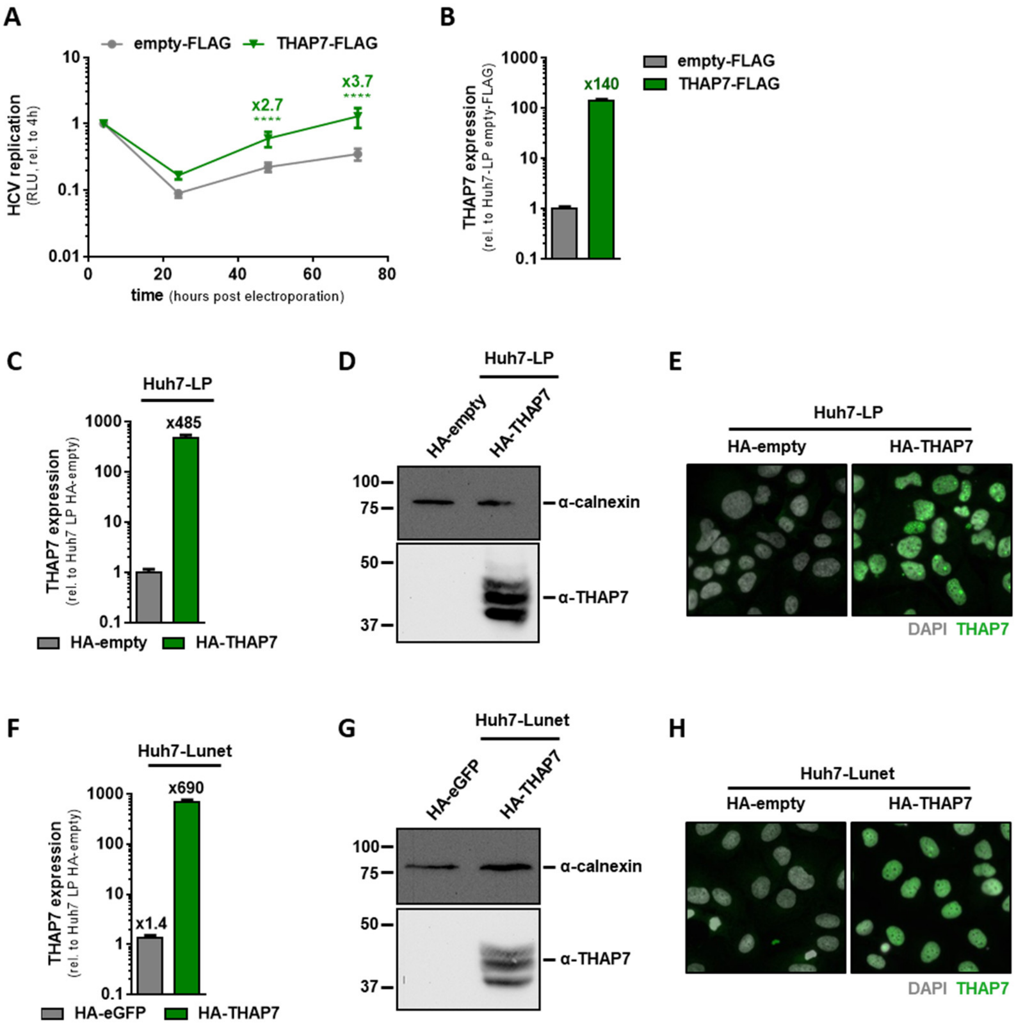

Figure S5. Overexpression of THAP7-FLAG in lowly permissive Huh7-LP increases HCV replication (A) and HA-THAP7 is robustly overexpressed in Huh7-LP and -Lunet cells (B-H). (**A**) HCV replication in Huh7-LP cells overexpressing THAP7-FLAG or an empty control (empty-FLAG) after electro-transfection of a subgenomic HCV luciferase reporter genome (gt1b, Con1-ET) in a time-course of 72h. (**B**) Overexpression control of THAP7-FLAG compared to an empty control (empty-FLAG) by qRT-PCR. (**C**, **D**, **E**) Overexpression control of HA-THAP7 versus an HA-empty vector control in lowly permissive Huh7-LP cells using qRT-PCR (**C**), immunoblot (**D**) or immunofluorescence (**E**). (**F**, **G**, **H**) Overexpression control of HA-THAP7 versus an HA-eGFP control in highly permissive Huh7-Lunet cells using qRT-PCR (**F**), immunoblot (**G**) or immuno-fluorescence (**H**). Luciferase data (**A**) is normalized to the signal four hours post electro-transfection and shows mean and standard deviation from three independent biological experiments. Significance was calculated using two-tailed, non-paired Student's *t*-test. **** p≤0.0001.

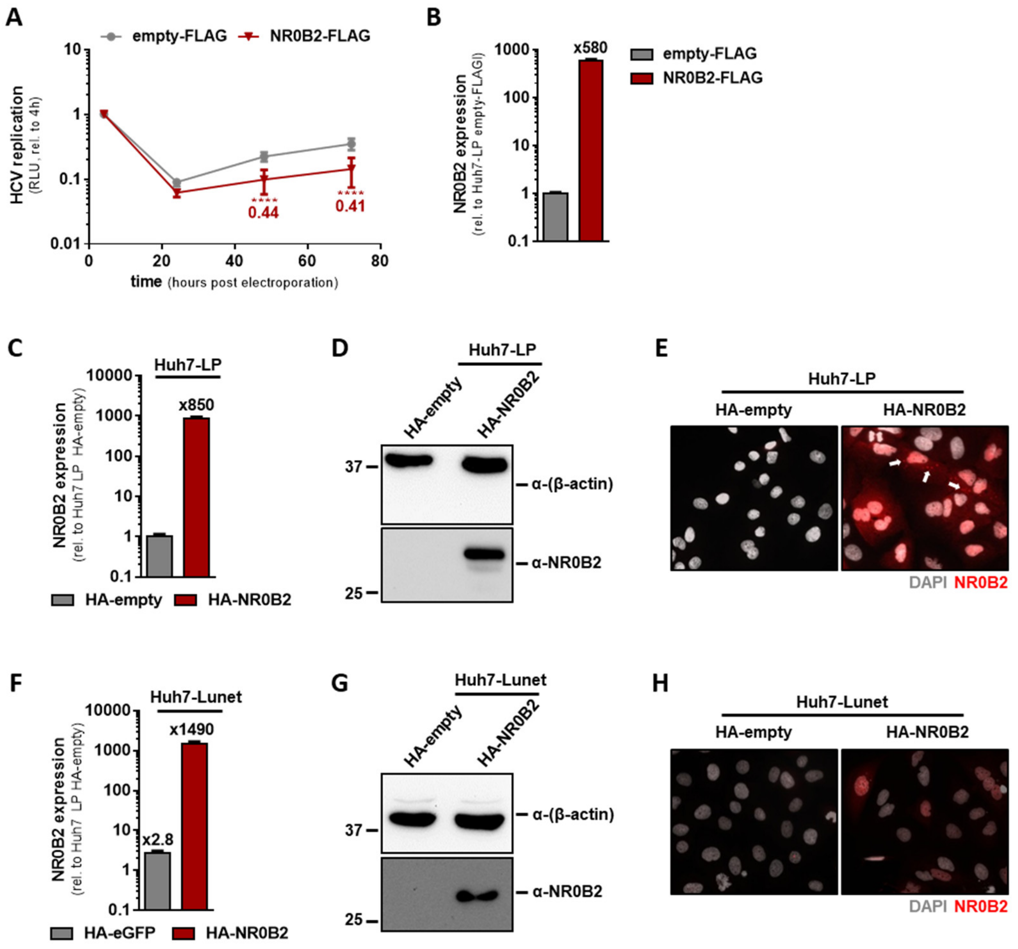

Figure S6. Overexpression of NR0B2-FLAG in lowly permissive Huh7-LP decreases HCV replication (A) and HA-NR0B2 is robustly overexpressed in Huh7-LP and -Lunet cells (B-H). (**A**) HCV replication in Huh7-LP cells overexpressing NR0B2-FLAG or an empty control (empty-FLAG) after electro-transfection of a subgenomic HCV luciferase reporter genome (gt1b, Con1-ET) in a time-course of 72h. (**B**) Overexpression control of NR0B2-FLAG compared to an empty control (empty-FLAG) by qPCR. (**C**, **D**, **E**) Overexpression control of HA-NR0B2 versus an HA-empty vector control in lowly permissive Huh7-LP cells using qRT-PCR (**C**), immunoblot (**D**) or immunofluorescence (**E**). (**F**, **G**, **H**) Overexpression control of HA-NR0B2 versus an HA-eGFP control in highly permissive Huh7-Lunet cells using qRT-PCR (**F**), immunoblot (**G**) or immuno-fluorescence (**H**). Luciferase data (**A**) is normalized to the signal four hours post electro-transfection and shows mean and standard deviation from three independent biological experiments. Significance was calculated using two-tailed, non-paired Student's *t*-test. **** p≤0.0001.

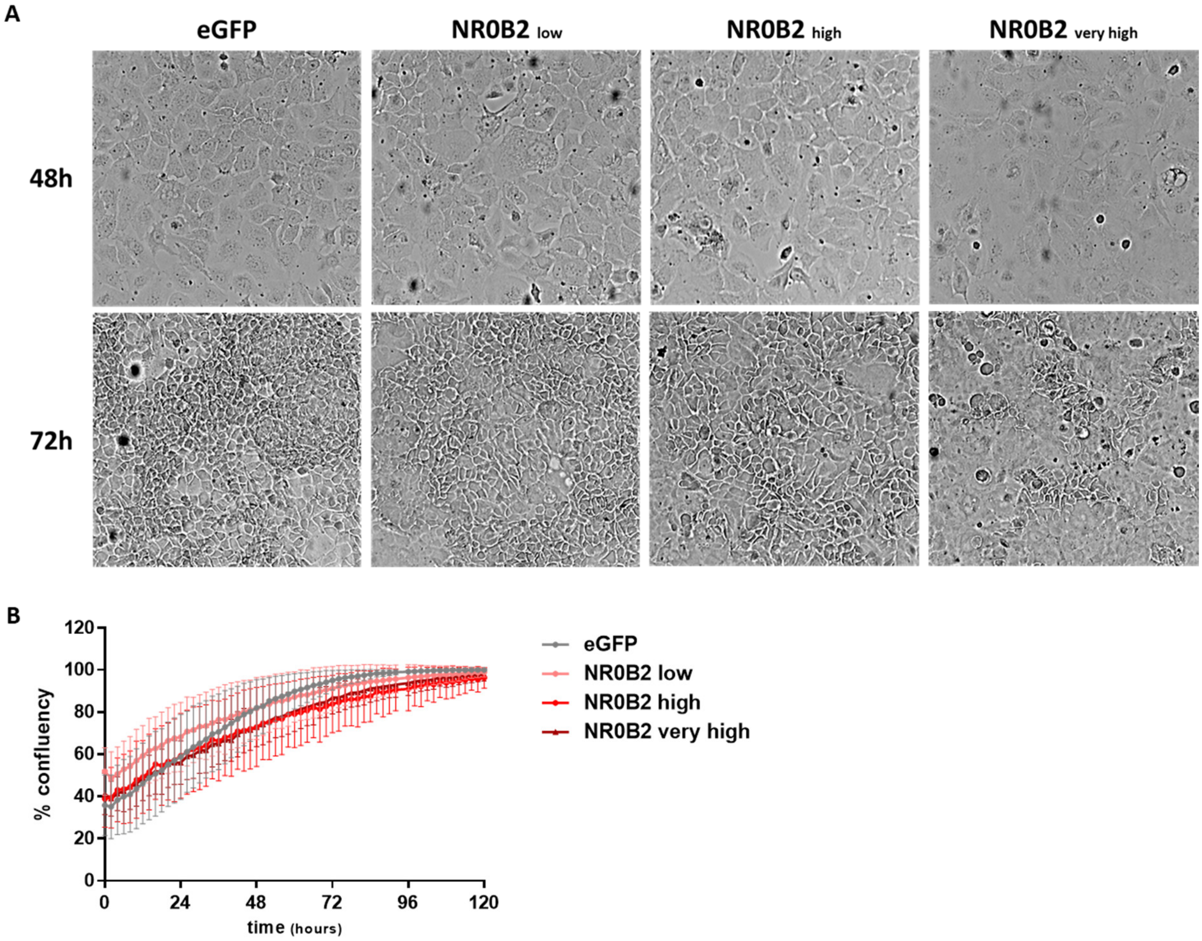

Figure S7. Huh7-Lunet cells overexpressing different amounts of NR0B2 show similar morphology (A) and growth kinetics (B). (**A**) Huh7-Lunet cells overexpressing eGFP or low, high, or very high amounts of NR0B2 were seeded on cover slips, fixed after 48 or 72 hours and imaged. (**B**) Cells were seeded on 96-well plates and cell growth was recorded by live cell imaging using the IncuCyte system.

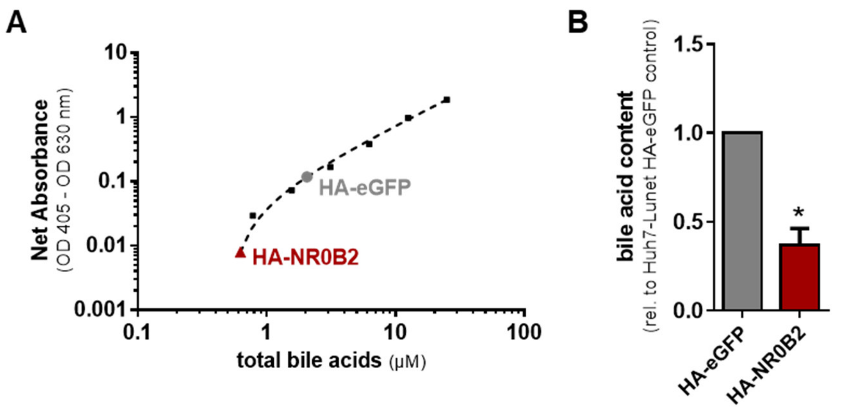

Figure S8. NR0B2 overexpression reduces the bile acid content in Huh7-Lunet cells. (**A**) Total bile acids from cell lysates were measured using the Total Bile Acid Assay kit (Cell Biolabs). Black squares represent standard samples and the dotted line is a linear regression of standard samples. (**B**) Quantification of total bile acid levels in Huh7-Lunet HA-NR0B2 overexpressing compared to HA-empty control cells. Data is normalized to the HA-eGFP control and shown as mean with standard deviation from two independent biological experiments. Significance was calculated using two-tailed, non-paired Student's *t*-test. * p≤0.05.

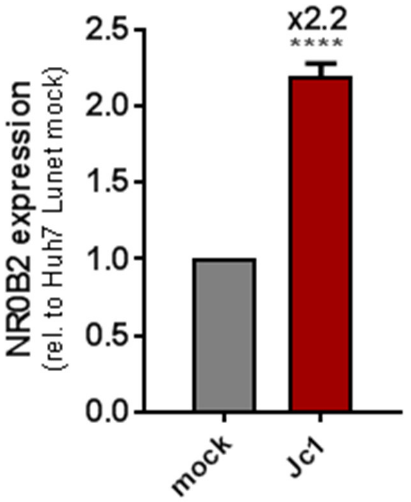

Figure S9. NR0B2 levels increase upon infection of Huh7-Lunet cells with cell culture-derived HCV (Jc1). Huh7-Lunet cells were infected with cell culture-derived HCV (Jc1) for 72h and NR0B2 expression was determined via qPCR. Data is normalized to the mock-infected control or the 0h time-point and shown as mean with standard deviation from three independent biological experiments. Significance was calculated using two-tailed, non-paired Student's *t*-test. **** $p \leq 0.0001$.

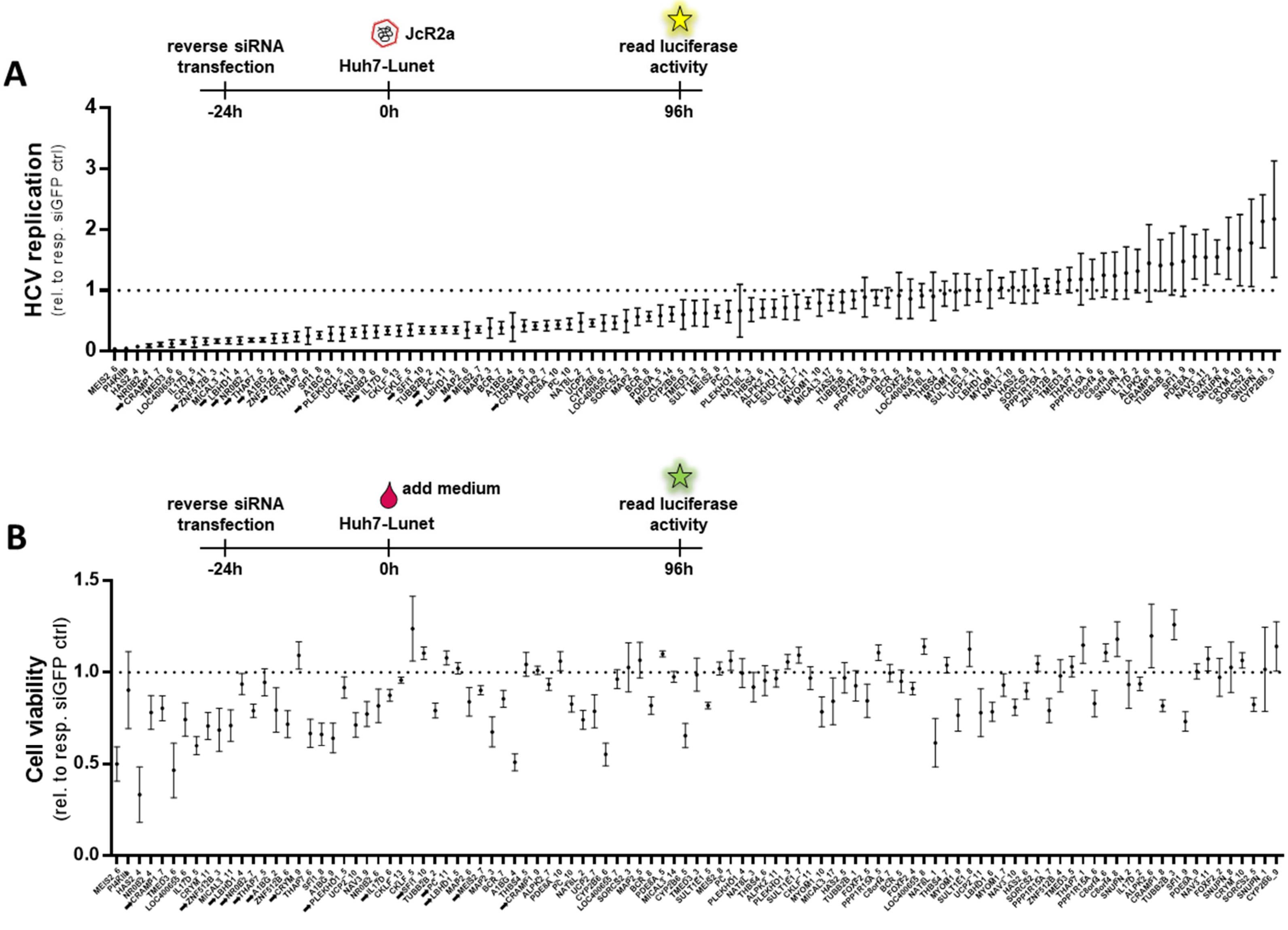

Figure S10. Effect of siRNA-mediated knockdown of potential host factors on HCV replication (A) and cell viability (B) in high permissive Huh7-Lunet cells. (**A**) Huh7-Lunet cells were reverse transfected with siRNAs against target genes (three siRNAs per gene) 24 hours before infection with the *Renilla* luciferase reporter virus JcR2a (gt2a) for 72 hours. Z-scores were calculated using a control siRNA targeting GFP for each experiment individually. Shown are means and standard deviations from the results of three independent biological experiments. (**B**) Huh7-Lunet cells were reverse transfected with siRNAs against target genes (three siRNAs per gene) 24 hours before mock infection and cell viability was measured 72 hours later. Data is normalized to a control siRNA targeting GFP for each experiment individually. Shown are means and standard deviations from two independent biological experiments. Arrows indicate selected hits that are shown in Figures 2 & S3.

Table S1. Effect of siRNAs used in the initial screening round on HCV replication and cell viability (see also Figure 2A). SiRNAs further used in a validation round on Huh7-LucUbiNeo cells are in bold and highlighted by gray background (see also Figure 2B).

		HCV replication		cell viability	
#	siRNA	mean	SD	mean	SD
1	MEIS2_6	0,04	0,01	0,50	0,09
2	**PI4KIIIα**	0,05	0,01	0,90	0,21
3	HAS2_4	0,08	0,03	0,33	0,15
4	NR0B2_4	0,09	0,03	0,78	0,09
5	**CRAMP1_7**	0,12	0,04	0,81	0,07
6	TMED3_6	0,13	0,07	0,46	0,15
7	LOC400655_6	0,15	0,04	0,74	0,09
8	IL17D_5	0,15	0,09	0,60	0,05
9	CRYM_11	0,16	0,06	0,71	0,07
10	**ZNF512B_3**	0,17	0,04	0,69	0,12
11	MICAL3_11	0,17	0,05	0,71	0,09
12	**LBHD1_4**	0,18	0,06	0,94	0,06
13	**NR0B2_7**	0,19	0,03	0,79	0,04
14	**THAP7_5**	0,19	0,04	0,95	0,07
15	**A1BG_2**	0,21	0,08	0,80	0,12
16	ZNF512B_6	0,22	0,08	0,72	0,07
17	CRYM_9	0,25	0,08	1,09	0,08
18	THAP7_6	0,25	0,14	0,67	0,08
19	SFI1_8	0,27	0,07	0,66	0,06
20	A1BG_9	0,29	0,12	0,64	0,08
21	**PLEKHO1_5**	0,28	0,12	0,92	0,06
22	UCP2_10	0,31	0,08	0,71	0,07
23	NAV3_9	0,32	0,11	0,77	0,07
24	NR0B2_6	0,32	0,10	0,82	0,09
25	**IL17D_6**	0,34	0,07	0,88	0,03
26	CKLF_13	0,34	0,09	0,96	0,02
27	CKLF_5	0,35	0,10	1,24	0,18
28	**SFI1_10**	0,35	0,06	1,11	0,04
29	TUBB2B_2	0,35	0,06	0,79	0,04
30	**PC_11**	0,35	0,06	1,08	0,04
31	**LBHD1_5**	0,35	0,07	1,02	0,03
32	MAP2_6	0,35	0,14	0,84	0,08
33	**MEIS2_7**	0,36	0,06	0,90	0,02
34	MAP2_3	0,38	0,17	0,68	0,08
35	BCR_7	0,38	0,11	0,86	0,05
36	A1BG_4	0,40	0,24	0,51	0,05
37	THBS4_5	0,42	0,10	1,04	0,07
38	**CRAMP1_9**	0,42	0,06	1,01	0,02
39	ALPK2_7	0,42	0,09	0,94	0,03
40	PDE8A_10	0,44	0,07	1,06	0,05
41	PC_10	0,45	0,10	0,83	0,04
42	NAT8L_2	0,47	0,16	0,74	0,05
43	UCP2_7	0,47	0,06	0,79	0,09
44	CYP2B6_6	0,47	0,14	0,55	0,06
45	LOC400655_7	0,47	0,11	0,96	0,05
46	SORCS2_3	0,50	0,19	1,03	0,13
47	MAP2_5	0,57	0,14	1,07	0,10
48	BCR_8	0,57	0,08	0,82	0,05
49	PDE8A_5	0,58	0,17	1,10	0,02
50	MICAL3_14	0,61	0,12	0,98	0,03
51	CYP2B6_5	0,60	0,24	0,66	0,07
52	TMED3_3	0,63	0,21	0,99	0,09
53	SULT1E1_5	0,62	0,22	0,82	0,02
54	MEIS2_8	0,65	0,11	1,02	0,04
55	PC_7	0,65	0,18	1,06	0,05
56	PLEKHO1_4	0,67	0,44	1,00	0,08
57	NAT8L_3	0,68	0,20	0,92	0,08
58	THBS4_6	0,70	0,15	0,96	0,08
59	ALPK2_11	0,71	0,15	0,97	0,05
60	PLEKHO1_3	0,72	0,19	1,06	0,04
61	SULT1E1_7	0,73	0,21	1,09	0,04
62	CKLF_11	0,79	0,09	0,97	0,06
63	MYOM1_10	0,79	0,22	0,79	0,08
64	MICAL3_17	0,80	0,13	0,84	0,13
65	HAS2_5	0,81	0,17	0,97	0,08
66	TUBB2B_1	0,85	0,14	0,93	0,08
67	FOXF2_5	0,89	0,32	0,85	0,09
68	PPP1R15A_5	0,88	0,13	1,11	0,04
69	C8orf4_7	0,88	0,17	1,00	0,05
70	BCR_5	0,92	0,38	0,95	0,06
71	FOXF2_4	0,86	0,33	0,91	0,03
72	LOC400655_8	0,92	0,20	1,14	0,04

		HCV replication		cell viability	
#	SD	mean	SD	mean	SD
73	NAT8L_1	0,90	0,40	0,62	0,13
74	THBS4_7	0,95	0,21	1,04	0,04
75	MYOM1_9	0,97	0,31	0,77	0,09
76	SULT1E1_8	1,01	0,26	1,13	0,09
77	UCP2_11	1,00	0,19	0,78	0,13
78	LBHD1_6	1,02	0,31	0,79	0,05
79	MYOM1_7	1,04	0,17	0,93	0,06
80	NAV3_10	1,05	0,25	0,81	0,04
81	HAS2_6	1,06	0,28	0,90	0,05
82	SORCS2_1	1,08	0,29	1,05	0,04
83	PPP1R15A_7	1,07	0,12	0,79	0,07
84	ZNF512B_4	1,14	0,20	0,98	0,09
85	TMED3_5	1,17	0,21	1,03	0,06
86	THAP7_1	1,18	0,42	1,15	0,10
87	PPP1R15A_6	1,18	0,33	0,83	0,07
88	C8orf4_6	1,25	0,36	1,11	0,05
89	C8orf4_8	1,24	0,39	1,18	0,10
90	SNUPN_2	1,29	0,43	0,93	0,13
91	IL17D_2	1,32	0,36	0,94	0,04
92	ALPK2_6	1,44	0,64	1,20	0,17
93	CRAMP1_8	1,41	0,42	0,82	0,03
94	TUBB2B_3	1,43	0,51	1,26	0,08
95	SFI1_9	1,48	0,58	0,73	0,05
96	PDE8A_9	1,55	0,37	1,01	0,04
97	NAV3_11	1,55	0,45	1,07	0,07
98	FOXF2_2	1,55	0,28	0,97	0,10
99	SNUPN_8	1,69	0,51	1,03	0,14
100	CRYM_10	1,66	0,58	1,07	0,04
101	SORCS2_5	1,78	0,72	0,82	0,04
102	SNUPN_1	2,14	0,44	1,02	0,23
103	CYP2B6_9	2,18	0,96	1,14	0,13
